# Real-world heart rate norms in the Health eHeart study

**DOI:** 10.1038/s41746-019-0134-9

**Published:** 2019-06-25

**Authors:** Robert Avram, Geoffrey H. Tison, Kirstin Aschbacher, Peter Kuhar, Eric Vittinghoff, Michael Butzner, Ryan Runge, Nancy Wu, Mark J. Pletcher, Gregory M. Marcus, Jeffrey Olgin

**Affiliations:** 1 Division of Cardiology, Department of Medicine, and the Cardiovascular Research Institute, University of California, San Francisco, Cardiology (San Francisco, CA, United States), 505 Parnassus Avenue, San Francisco, CA 94143 USA; 2 Azumio, inc (Palo Alto, CA, United States), 145, 255 Shoreline Drive, Redwood City, CA 94065 USA; 3 Department of Epidemiology and Biostatistics, University of California San Francisco (San Francisco, CA, United States), 505 Parnassus Avenue, San Francisco, CA 94143 USA

**Keywords:** Epidemiology, Predictive markers

## Abstract

Emerging technology allows patients to measure and record their heart rate (HR) remotely by photoplethysmography (PPG) using smart devices like smartphones. However, the validity and expected distribution of such measurements are unclear, making it difficult for physicians to help patients interpret real-world, remote and on-demand HR measurements. Our goal was to validate HR-PPG, measured using a smartphone app, against HR-electrocardiogram (ECG) measurements and describe out-of-clinic, real-world, HR-PPG values according to age, demographics, body mass index, physical activity level, and disease. To validate the measurements, we obtained simultaneous HR-PPG and HR-ECG in 50 consecutive patients at our cardiology clinic. We then used data from participants enrolled in the Health eHeart cohort between 1 April 2014 and 30 April 2018 to derive real-world norms of HR-PPG according to demographics and medical conditions. HR-PPG and HR-ECG were highly correlated (Intraclass correlation = 0.90). A total of 66,788 Health eHeart Study participants contributed 3,144,332 HR-PPG measurements. The mean real-world HR was 79.1 bpm ± 14.5. The 95th percentile of real-world HR was ≤110 in individuals aged 18–45, ≤100 in those aged 45–60 and ≤95 bpm in individuals older than 60 years old. In multivariable linear regression, the number of medical conditions, female gender, increasing body mass index, and being Hispanic was associated with an increased HR, whereas increasing age was associated with a reduced HR. Our study provides the largest real-world norms for remotely obtained, real-world HR according to various strata and they may help physicians interpret and engage with patients presenting such data.

## Introduction

Heart rate (HR) is a readily available vital sign that holds important prognostic information. Generally, lower HR has been associated with lower all-cause and cardiovascular mortality.^1–5^ Several studies, as well as expert consensus, indicate that the normal adult resting HR values lie between 60 and 90 beats per minute (bpm),^1–3^ and the American Heart Association defines the normal sinus HR as between 60 and 100 bpm.^3^ However, these commonly accepted norms are derived using in-clinic recorded HR which may not be representative of the real-world, outside of a healthcare institution, remotely obtained measurements that are commonly recorded by a growing number of consumer devices. For example, clinic measured data can be artificially increased in a similar phenomenon to “white-coat hypertension”^4^ or by an increased adrenergic reaction to the clinical settings.^5^ In addition, these measurements do not account for health status, cardiovascular fitness, gender, or racial differences. Moreover, ambulatory heart rate has been found to be a stronger predictor for all-cause mortality than in-clinic resting heart rate, yet this real-world measurement is infrequently obtained.^6^

Recently, photoplethysmography (PPG) technology has become nearly ubiquitous in smartphones and wearable sensors (such as activity trackers or smartwatches), providing both an opportunity to measure real-world HR while increasing the importance to understand the accuracy and the normal HR values obtained by these types of ambulatory measurement.^7,8^ In addition, physicians are increasingly being asked by patients to interpret HR values recorded remotely by patient devices.^9^ However, in this setting it is unclear whether traditional clinic-derived normal values adequately represent remotely recorded real-world data.^9^

The Health eHeart Study, an online Framingham-like cohort, has collected a large number of HR measurements over time from study participants using PPG-enabled smartphone technology. The goals of this study were to (i) validate HR-PPG measurements against a gold-standard electrocardiographic HR-electrocardiogram (ECG) measurement, and (ii) provide real-world HR-PPG ranges according to age, time, demographics, comorbidities and chronotropic medication usage, and (iii) identify predictors of real-world HR-PPG and heart rate variability (HRV).

## Results

### Smartphone-based PPG validation

We validated the HR-PPG measurement in 50 consecutive participants seen at the UCSF general cardiology clinic who had a 12-lead ECG performed (10 s recording) with simultaneous PPG signals recorded (Supplementary Figs. 1 and 2). These patients were older 64.0 ± 13.1 (vs 43.4 ± 14.8 in our full HR data set; *p* < 0.0005), male (66.0% vs 52.3%; *p* < 0.00005) and they had a higher prevalence of diabetes, hypercholesterolemia, hypertension, and arrhythmia than that in our full HR data set. There were 21 abnormal ECGs (five atrial fibrillation, two atrial flutter, three left bundle branch block, three frequent premature ventricular complex, one frequent premature atrial complex, one sinus tachycardia, six ventricular pacemaker) and 29 normal ECGs with normal sinus rhythm. The HR-PPG values had very good intraclass correlation (ICC) with HR-PPG (0.90 overall; 0.88 for irregular rhythms; and 1.00 for regular rhythms) with a median absolute HR difference between both recordings of 2.7 bpm (6.9) (Supplementary Fig. 3A). The median difference between the two signals in successive R–R interval measurements was 12.5 ms (23.4) and the ICC between signals was very high (1.00 overall, 0.99 for irregular rhythms and 1.00 for regular rhythms) (Supplementary Fig. 3B). The Bland–Altman plots showed no evidence of trends in either the bias or the dispersion of the differences, at low, normal, or high HR values.

### Health eHeart study sample

A total of 66,788 Health eHeart Study participants contributed 3,144,332 HR-PPG measurements between April 1 2014 and 30 April 2018, forming our “full HR data set”. Of these, 33,344 (1.06%) measurements were excluded for being outside of biological ranges (Supplementary Fig. 4). In our full HR data set, mean age was 43.3 ± 14.8 years and 47.0% of our participants were female (Table 1). The BMI was 27.5 ± 5.8 kg/m^2^ and participants walked on average 3491.1 ± 3345.4 steps per day, as measured by their smartphone. Slightly less than half of the participants were healthy, having reported no medical condition (*n* = 25,408, 48.3%). The most prevalent medical conditions were hypertension, hypercholesterolemia and presence of arrhythmia (Table 1). In addition, 2412 (6.9%) users were treated with beta blockers and 435 (1.3%) were on non-dihydropyridine CCBs, amiodarone, or inhaled beta agonists (Table 1).

**Table 1:** Baseline characteristics

Baseline characteristics	No reported medical conditions *N*, (%) *N* = 25,408^a^	Individuals with at least one medical condition *N*, (%) *N* = 27,958^a^	Full HR data set^a^ *n*, (%) *n* = 66,788	Validation cohort *n*, (%) *n* = 50
Age, mean ± SD, yrs	37.7 ± 13.0	47.7 + 14.5	43.4 ± 14.8	64.0 ± 13.1
Number of HR-PPG values recorded	1,101,550	1,506,946	3,110,988	–
Number of HR-PPG measurements per user, per year, median (IQR)	59.7 (143.4)	65.2 (154.4)	60.1 (145.4)	1 (0)
Geometric HR-PPG, mean ± SD, bpm	77.6 ± 14.6	79.6 ± 14.2	79.1 ± 14.5	–
Demographics	*N* = 18,858	*N* = 18,402	*N* = 37,240	*N* = 50
Females	9476 (50.2)	8063 (43.8)	17,519 (47.0)	17 (34.0)
Males	9382 (49.8)	10,339 (56.2)	19,721 (52.3)	33 (66.0)
Race or ethnic group	*N* = 18,727	*N* = 18,168	*N* = 37,258	–
Non-Hispanic White	14,280 (76.3)	13,790 (75.9)	28,351 (76.1)	–
Black or African	314 (1.7)	486 (2.7)	813 (2.2)	–
American Hispanic, Latino, or Spanish origin or ancestry	2001 (10.7)	1992 (11.0)	4023 (10.7)	–
Asian	1160 (6.2)	1028 (5.7)	2212 (5.9)	–
Multi-ethnic	526 (2.8)	489 (2.7)	1022 (2.7)	–
Other	446 (2.4)	383 (2.1)	837 (2.2)	–
Anthropometric data	*N* = 2175	*N* = 2586	*N* = 6025	*N* = 50
Height, mean ± SD, m	1.73 ± 0.10	1.73 ± 0.10	1.73 ± 0.10	1.74 ± 0.11
Weight, mean ± SD, kg	77.9 ± 17.8	86.1 ± 20.8	82.5 ± 19.9	85.8 ± 22.2
BMI, mean ± SD, kg/m^2^	26.1 ± 5.3	28.7 ± 6.0	27.5 ± 5.8	28.2 ± 5.3
Physical activity data	*N* = 5343	*N* = 4,810	*N* = 14,216	
Average daily step count, mean ± SD, steps	3793.8 ± 3,504.6	3244 ± 3,220	3491.1 ± 3345.4	–
Past medical history	*N* = 25,408	*N* = 27,958	*N* = 53,366 †	*N* = 50
No reported medical conditions	25,408 (100)	–	25,408 (48.3)	25 (50.0)
Essential hypertension	–	13,939 (49.9)	13,939 (26.5)	22 (44.0)
Hypercholesterolemia	–	15,088 (54.0)	15,088 (28.7)	18 (36.0)
Diabetes	–	3505 (12.5)	3505 (6.7)	4 (8.0)
CAD	–	3699 (13.2)	3699 (7.0)	0 (0)
Prior MI	–	1791 (6.4)	1791 (3.4)	0 (0)
Arrhythmia	–	7560 (27.0)	7560 (14.3)	11 (22.0)
CHF	–	1172 (4.2)	1172 (2.3)	4 (8.0)
PVD	–	1027 (3.7)	1027 (2.0)	0 (0)
Prior stroke	–	1458 (5.2)	1458 (2.9)	0 (0)
Sleep Apnea	–	6923 (24.8)	6923 (13.2)	0 (0)
Asthma	–	5622 (20.1)	5622 (10.7)	0 (0)
COPD	–	1685 (6.0)	1685 (3.2)	0 (0)
Medications	–	*N* = 35,074	*N* = 35,074	–
Beta blockers	–	2412 (6.8)	2412 (6.8)	–
Non-dihydropyridine CCB	–	260 (0.7)	260 (0.7)	–
Amiodarone	–	55 (0.2)	55 (0.2)	–
Inhaled beta agonists	–	120 (0.3)	120 (0.3)	–

*BMI* body mass index, *CAD* coronary artery disease, *kg* kilogram, *m* meter, *cm* centimeters, *CHF* congestive heart failure, *COPD* chronic obstructive pulmonary disease, *HR* heart rate, *MI* myocardial infarction, *PPG* photoplethysmography, *PVD* peripheral vascular disease, *SD* standard deviation

^a^The distribution of all variables between the “no reported medical conditions” data set, the “Individuals with at least one medical condition” data set and the “full HR” data set are significantly different (*p* < 0.0005), except the “height” (*p* = 0.65) and the average daily step counts (*p* = 0.57) and race/ethnic group (*p* = 0.01)

### Description of our HR data sets

A total of 40,572 measurements from 8046 participants met our definition for inclusion in the “known resting HR data set”. After obtaining real-world user-specific HR-PPG, the geometric mean HR-PPG in our “known resting HR” data set was 2.8 bpm higher compared with our “full HR data set” and had a higher spread of values (81.8 ± 19.6 (95% percentile interval: 52.5–132.1) vs 79.0 ± 14.5 (95% percentile interval: 54.5–110.8), respectively; *p* < 0.0005 (Supplementary Tables 2A–C, 4 and Fig. 5).

In our “full HR data set”, real-world HR varied significantly over the day with the lowest values observed between midnight and 5 AM (nadir at 5 am; 75.8 ± 22.4) and the highest values observed between 5 AM and 5 PM (peak at 5 pm; 82.3 ± 23.7; *p* < 0.0005) (Supplementary Fig. 6). The HR was higher during weekdays compared with weekends (79.1 ± 17.6 vs. 78.4 ± 17.1; *p* < 0.0005). Similarly, the HRV was highest between 6 am and 12 pm (14.9 ± 10.6) and lowest between 6 pm and 12 am (12.5 ± 9.6; *p* < 0.0005 compared with 6 am–12 pm). It was also higher during weekdays than weekends (13.7 ± 10.7 vs. 13.3 ± 10.4; *p* < 0.0005) (Supplemental Table 1). Finally, the highest average HR was observed during winter (79.1 ± 16.7), whereas the lowest HR was observed during fall (78.4 ± 16.5; *p* < 0.0005 compared with winter). HRV was highest during summer (15.0 ± 10.2) and fell to the lowest levels during winter (14.1 ± 10.0; *p* < 0.0005 compared with summer) and spring (14.1 ± 10.2; *p* < 0.0005 compared with summer).

### Heart rate according to age, demographics, step count, comorbidities, and medications

**Fig. 1:**
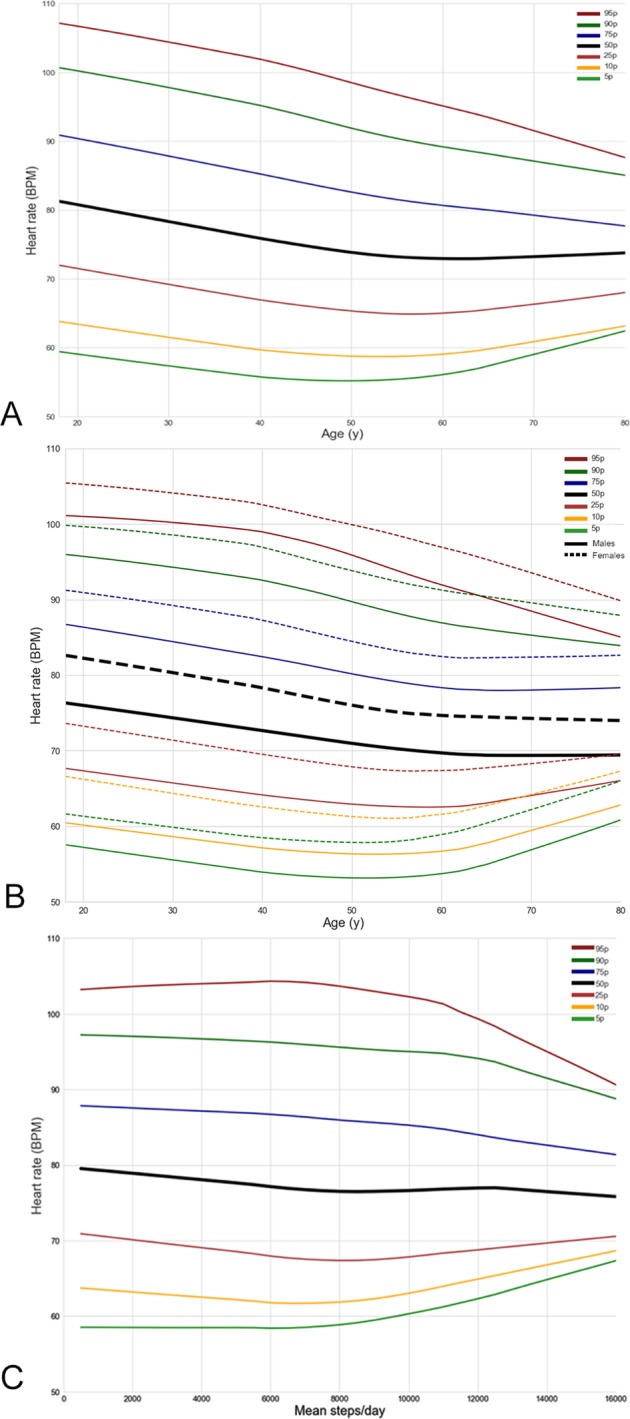
Percentile graph of average real-world HR-PPG. **a** Percentile graph of average real-world HR-PPG according to the age. **b** Percentile graph of average real-world HR-PPG according to the gender **c** Percentile graph of average real-world HR-PPG according to the step counts

We describe variations in HR-PPG according to various factors within the subgroup who reported no medical conditions (*n* = 25,408; 48.3% of users and *n* = 1,103,570 measurements). These “healthy individuals” were younger (37.7 ± 13.0 vs. 43.4 ± 14.8; *p* < 0.0005), with a higher proportion of females (50.2% vs 47.0%; *p* < 0.0005), similar racial/ethnic group composition, similar step counts and a lower BMI (26.1 ± 5.3 vs. 28.7 ± 6.0) when compared with our “full HR data set”. In healthy individuals, average resting HR-PPG decreased from 81.6 ± 14.0 in those aged 18–20 to 74.2 ± 12.7 in those aged 71–80 (*p* < 0.0005) (Table 2 and Fig. 1a). The 95th percentile of real-world HR was uniformly under 100 bpm after 45 years of age reaching approximately 95 bpm at 61 years of age. Females had on average a HR-PPG 4.4 bpm higher than men (Fig. 1b). As age increased, the 95% CI of HR-PPG values narrowed in women more than men (18–20 years, women 56.8–113.1 bpm, men 54.4–106.2 bpm; vs age 60–70, women 56.3–103.7 bpm, men 52.4–101.9 bpm, Fig. 1b and Supplementary Table 2B, C). African Americans and Asians had the highest HR-PPG (81.4 ± 14.0 bpm and 79.2 ± 14.3 bpm, respectively) and non-Hispanic White had the lowest HR-PPG (75.9 ± 14.5 bpm) (Table 2). HR-PPG increased with BMI from 74.9 ± 16.5 bpm in individuals with a BMI of 18.5–25 kg/m^2^ to 80.1 ± 13.3 bpm in those with BMI ≥ 30 kg/m^2^ (*p* < 0.0005). A negative relationship between HR-PPG and daily step counts was observed, with the HR-PPG values significantly decreasing from 80.0 ± 13.5 bpm in sedentary participants walking 2001–4000 steps per day to 78.0 ± 13.9 bpm in active participants walking 8001–12,000 steps per day (*p* < 0.0005) (Table 2, Fig. 1c and Supplementary Table 3). We observed no significant difference in the real-world HR-PPG between groups above an average of 8001 steps per day. Height was a predictor of reduced heart rate where for every 1 centimeter, the HR was reduced by 0.23 bpm. In univariable analysis, age, height, and number of steps were negative predictors of heart rate and female gender, BMI, Asian race and multi-ethnicity were predictors of an increased heart rate (Supplementary Table 5). Weight was not a significant predictor of heart rate. In a multivariable analysis, age was significantly associated with lowered heart rate, whereas female gender, BMI, Hispanic ethnicity and the number of medical conditions had a positive relationship with HR-PPG, however step counts were not a significant independent predictor of HR-PPG (Table 4; Model 1).

**Table 2:** Heart rate according to age, gender, race, and step count in healthy participants

Baseline characteristics	Number of participants contributing at least one HR-PPG measurement *N*, (%)	Number of HR-PPG measurements Median (IQR)	Geometric mean HR ± SD^a^
Age stratum	*N* = 25,280		
18–20	2197 (8.7)	11.0 (20.0)	81.6 ± 14.0
21–30	6558 (25.9)	15.0 (30.0)	80.2 ± 14.8
31–40	6480 (25.6)	18.0 (37.0)	78.5 ± 15.1
41–50	5620 (22.2)	21.0 (45.0)	75.3 ± 14.3
51–60	3058 (12.1)	23.0 (54.0)	73.9 ± 13.5
61–70	1173 (4.6)	26.0 (54.0)	73.0 ± 12.7
71–80	194 (0.8)	29.5 (72.3)	74.2 ± 11.1
>80	13 (0.1)	36.0 (80.0)	78.1 ± 16.5
*Gender*	*N* = 18,858		
Females	9476 (50.2)	18.0 (38)	79.3 ± 14.0
Males	9382 (49.8)	21.0 (45)	73.8 ± 14.5
Race or ethnic group	*N* = 18,727		
Non-Hispanic White	14,280 (76.3)	20.0 (43.0)	75.9 ± 14.5
Black or African American	314 (1.7)	12.0 (36.8)	81.4 ± 14.0
Hispanic, Latino, or Spanish origin or ancestry	2001 (10.7)	18.0 (37.0)	78.6 ± 14.3
Asian	1160 (6.2)	17.0 (38.8)	79.2 ± 14.3
Multi-ethnic	526 (2.8)	20.0 (34.0)	78.1 ± 14.4
Other/prefer not to disclose	440 (2.4)	15 (34.0)	78.2 + 13.0
BMI	*N* = 2175		
<18.5	58	21.0 (54.2)	77.9 ± 14.7
≥18.5–25	1027	29.0 (62.0)	74.9 ± 16.5
25–30	686	29.0 (69.0)	73.0 ± 15.1
≥30	404	24.5 (58.8)	80.1 ± 13.3
Daily step count stratum	*N* = 5343		
100–2000	3646 (68.2)	36.0 (66.0)	78.9 ± 14.4
2001–4000	826 (15.4)	35.0 (72.0)	80.0 ± 13.5
4001–6000	422 (7.9)	39.5 (82.0)	79.0 ± 14.8
6001–8000	241 (4.5)	31.0 (77.0)	77.6 ± 16.4
8001–10,000	113 (2.1)	45.0 (80.0)	77.9 ± 17.3
10,001–12,000	64 (1.2)	23.0 (69.3)	78.0 ± 13.9
12,001–14,000	31 (0.6)	31.0 (37.0)	81.9 ± 15.0

*HR-PPG* heart rate as measured using photoplethysmography, *BMI* body mass index, *HR* heart rate, *SD* standard deviation

^a^All intergroup comparisons were significant (*p* < 0.0005)

**Table 3:** Heart rate according to past medical history and medication

Baseline characteristics	Number of participants contributing at least one measurement *n* (%)	Number of measurements per participant median (IQR)	Unadjusted geometric mean HR-PPG ± SD (bpm)	*P* value for unadjusted HR-PPG	Age-adjusted geometric mean HR-PPG ± SD (bpm)	*P* value for age-adjusted means
Self-reported past medical history	*N* = 53,366					
No medical condition	25,408 (47.6)	18.0 (38.0)	77.6 ± 14.6	<0.0005	75.5 ± 0.2	<0.0005
Presence of at least one medical condition	27,958 (52.4)	20.0 (47.0)	79.6 ± 14.2		79.5 ± 0.1	
Essential hypertension						
With	13,939 (26.5)	20.0 (49.0)	79.5 ± 14.1	0.0005	79.9 ± 0.2	<0.0005
Without	38,495 (73.5)	18.0 (42.0)	78.2 ± 14.6		76.7 ± 0.1	
Hypercholesterolemia						
With	15,088 (28.7)	20.0 (49.0)	78.8 ± 14.0	0.02	79.2 ± 0.2	<0.0005
Without	37, 346 (71.3)	18.0 (42.0)	78.4 ± 14.7		76.7 ± 0.1	
Diabetes						
With	3505 (6.7)	18.0 (46.0)	82.6 ± 14.1	<0.0005	83.7 ± 0.3	<0.0005
Without	49,635 (93.3)	19.0 (42.0)	78.3 ± 14.5		77.1 ± 0.1	
CAD						
With	3699 (7.0)	22.0 (55.5)	78.6 ± 14.4	0.77	78.9 ± 0.3	<0.0005
Without	49,331 (93.0)	18.0 (42.0)	78.5 ± 14.5		77.4 ± 0.1	
Prior MI						
With	1791 (3.4)	20.0 (52.0)	77.9 ± 14.4	0.03	78.5 ± 0.5	0.03
Without	51,575 (9.6)	18.0 (42.0)	78.6 ± 14.5		77.5 ± 0.1	
Arrhythmia						
With	7560 (14.3)	25.0 (59.0)	81.0 ± 15.3	<0.0005	79.9 ± 0.2	<0.0005
Without	44,867 (85.7)	18.0 (39.0)	78.0 ± 14.3		77.1 ± 0.1	
CHF						
With	1172 (2.3)	22.0 (59.0)	80.9 ± 13.9	<0.0005	80.6 ± 0.6	<0.0005
Without	52,194 (97.7)	19.0 (42.0)	78.5 ± 14.5		77.5 ± 0.1	
PVD						
With	1027 (2.0)	20.0 (52.5)	80.0 ± 14.1	<0.0005	80.0 ± 0.7	<0.0005
Without	52,339 (98.0)	19.0 (42.0)	78.5 ± 14.5		77.5 ± 0.1	
Prior stroke						
With	1458 (2.9)	21.0 (51.0)	80.4 ± 14,0	<0.0005	80.6 ± 0.5	<0.0005
Without	51,908 (96.1)	19.0 (42.0)	78.5 ± 14.4		77.4 ± 0.1	
Sleep apnea						
With	6923 (13.2)	19.0 (46.0)	81.0 ± 14.2	<0.0005	81.3 ± 0.2	<0.0005
Without	46,443 (86.8)	19.0 (42.0)	77.9 ± 14.5		76.9 ± 0.1	
Asthma						
With	5622 (10.7)	17.0 (40.0)	81.6 + 14.5	<0.0005	80.1 ± 0.2	<0.0005
Without	47,744 (89.3)	19.0 (43.0)	78.2 ± 14.4		77.2 ± 0.1	
COPD						
With	1685 (3.2)	18.0 (43.0)	82.5 ± 13.9	<0.0005	82.2 ± 0.5	<0.0005
Without	51,681 (96.8)	19.0 (43.0)	78.3 ± 14.5		77.4 ± 0.1	
Medications	*N* = 35,074					
Beta blockers						
Taking	2412 (6.9)	35.5 (86.0)	77.7 ± 13.3	0.96	78.6 ± 0.3	<0.0005
Non-taking	32,662 (93.1)	22.0 (47.0)	77.7 ± 14.5		77.0 ± 0.1	
Non-dihydropyridine CCB						
Taking	260 (0.7)	22.0 (49.0)	81.8 ± 14.6	<0.0005	83.3 ± 0.9	<0.0005
Non-taking	34,814 (99.3)	47.0 (97.5)	77.6 ± 14.5		77.1 ± 0.1	
Amiodarone						
Taking	55 (0.2)	48.0 (137.0)	74.4 ± 12.5	0.09	76.8 ± 1.9	0.84
Non-taking	35,019 (98.2)	22.0 (49.0)	77.7 ± 14.5		77.2 ± 0.1	
Inhaled beta agonists						
Taking	120 (0.3)	23.0 (60.8)	79.7 ± 18.4	0.04	81.3 ± 1.4	0.003
Non-taking	34,954 (97.7)	22.0 (49.0)	76.5 ± 18.5		77.2 ± 0.1	

*CAD* coronary artery disease, *CHF* congestive heart failure, *COPD* chronic obstructive pulmonary disease, *CCB* calcium channel blockers, *HR* heart rate, *MI* myocardial infarction, *PVD* peripheral vascular disease, *SD* standard deviation

^a^These values are only reported in “healthy” participants

**Table 4:** Multivariable regression models for mean heart rate

Model 1. Multivariable linear regression describing the relationship between age, gender, daily step count, body mass index and number of diseases with geometric mean HR-PPG, in 1400 participants^a^				Model 2. Multivariable linear regression describing the relationship between age, gender, disease state and medications with geometric mean HR-PPG, in 31,393 participants^b^			
Variable	Coefficient	95% CI	*p* value	Variable	Coefficient	95% CI	*p* value
Age, per 10-year increments	−2.59	−3.18–2.01	<0.0005	Age, per 10-year increments	−1.93	−2.05–1.81	<0.0005
Gender, for females	4.00	2.40–5.57	<0.0005	Gender, for females	4.28	3.97–4.59	<0.0005
Race or ethnic group			<0.0005	Race or ethnic group			<0.0005
Non-Hispanic White	Ref.	–	–	Non-Hispanic White	Ref.	–	
Black or African American	0.57	−4.21–5.55	0.79	Black or African American	3.00	1.94–4.06	<0.0005
Hispanic, Latino, or Spanish origin or ancestry	0.37	−2.42–3.17	0.79	Hispanic, Latino, or Spanish origin or ancestry	1.87	1.38–2.36	<0.0005
Asian	5.65	2.30–8.99	0.001	Asian	3.32	2.67–3.96	<0.0005
Multi-ethnic	−0.47	−4.98–4.03	0.84	Multi-ethnic	1.25	0.32–2.18	<0.0005
Other/prefer not to disclose	−0.02	−7.27–7.23	1.00	Other/prefer not to disclose	1.82	0.81–2.83	<0.0005
Number of medical conditions, per medical condition	0.83	0.22–1.44	0.01	Diabetes	4.48	3.78–5.19	<0.0005
Average daily steps, per 1000 steps	0.03	−0.19–0.25	0.79	Arrhythmia	1.65	1.19–2.11	<0.0005
BMI	0.21	0.08–0.34	0.001	Sleep apnea	3.67	3.19–4.16	<0.0005
				CCB, non-dihydropyridine	4.11	2.33–5.89	<0.0005
				COPD	2.49	1.50–3.48	<0.0005
				Asthma	1.51	0.99–2.03	<0.0005
				Hypertension	1.83	1.43–2.23	<0.0005
				Hypercholesterolemia	1.35	0.98–1.72	<0.0005
				Beta agonists	2.58	−0.10–5.26	0.06
				Beta blockers	−0.48	−1.12–0.16	0.14
				Amiodarone	−2.07	−5.95–1.81	0.30
				CAD	−0.28	−1.12–0.56	0.51
				Prior MI	−0.25	−1.43–0.94	0.68
				PVD	0.28	−1.20 −1.75	0.72
				Prior stroke	0.64	−0.43–1.71	0.24
				CHF	0.36	−1.05–1.77	0.62

*BMI* body mass Index, *CAD* coronary artery disease, *CCB* calcium channel blockers, non-dihydropyridine, *CHF* congestive heart failure, *CI* confidence interval, *COPD* chronic obstructive pulmonary disease, *HR* heart rate, *PPG* photoplethysmography, *PVD* peripheral vascular disease, *MI* myocardial Infarction, *y* years

^a^We had 1400 observations for model 1, owing to the inclusion of step counts and BMI as a predictor. The adjusted *R*^2^ was 0.01; *P* < 0.0005.

^b^We had 31,393 observations for model 2. The adjusted *R*^2^ was 0.09; *P* < 0.0005

An increase in average daily steps was associated with higher HRV, whereas an increase in age and BMI were significant predictors of a lower HRV (Table 5; Model 1). In Model 2, looking at age, gender, disease state, and medications, we observed a lower HRV for increasing age, female gender, hypertension, and an increase in HRV for Hispanic ethnicity, sleep apnea, and users on CCB (Table 5; Model 2). No other medical conditions or medications were significant predictors of HRV in our cohort.

**Table 5:** Multivariable regression models for intra-user standard deviation of heart rate measurements

Model 1. Multivariable linear regression describing the relationship between age, gender, daily step count, body mass index and number of diseases with geometric mean HR-PPG in 1652 participants^a^				Model 2. Multivariable linear regression describing the relationship between age, gender, disease state, and medications with intra-user standard deviation of HR-PPG, in 30,700 participants^b^			
Variable	Coefficient	95% CI	*p*-value	Variable	Coefficient	95% CI	*p*-value
Age, per 10-year increments	−0.47	−0.79–0.14	0.01	Age, per 10-year increments	−0.45	−0.53–0.37	<0.0005
Gender, for females	0.34	−0.54–1.28	0.45	Gender, for females	−0.48	−0.69–0.27	<0.0005
Race or ethnic group				Race or ethnic group			<0.0005
Non-Hispanic White	Ref.	–		Non-Hispanic White	Ref.	–	
Black or African American	−0.06	−2.77–2.64	0.96	Black or African American	0.61	−0.09–1.31	0.09
Hispanic, Latino, or Spanish origin or ancestry	0.28	−1.27–1.83	0.72	Hispanic, Latino, or Spanish origin or ancestry	0.65	0.33–0.98	<0.0005
Asian	0.17	−1.69–2.03	0.86	Asian	0.09	−0.34–0.52	0.67
Multi-ethnic	−0.85	−3.35–1.64	0.50	Multi-ethnic	0.19	−0.42–0.81	0.81
Other/prefer not to disclose	−0.60	−4.62–3.42	0.77	Other/prefer not to disclose	−0.31	−0.98–0.36	0.36
Number of medical conditions, per medical condition							
	0.07	−0.28–0.41	0.70	Diabetes	−0.28	−0.75–0.19	0.24
Average daily steps, per 1000 steps	0.15	0.03–0.28	0.02	Arrhythmia	0.89	0.59–1.19	<0.0005
BMI	−0.10	−0.18–0.03	0.01	Sleep apnea	0.63	0.30–0.95	<0.0005
				Non-dihydropyridine CCB	3.92	2.18–5.67	<0.0005
				COPD	−0.22	−0.88–0.43	0.50
				Asthma	−0.29	−0.63–0.05	0.10
				Hypertension	−0.47	−0.73–0.20	<0.0005
				Hypercholesterolemia	−0.08	−0.32–0.17	0.55
				Beta agonists	0.10	−1.65–1.86	0.91
				Beta blockers	0.002	−0.419–0.423	0.99
				Amiodarone	1.01	−1.61– 3.62	0.45
				CAD	−0.13	0.70–0.42	0.65
				Prior MI	0.26	−0.53–1.04	0.52
				PVD	0.45	−0.51–1.42	0.36
				Prior stroke	0.49	−0.22–1.20	0.17
				CHF	−0.24	−1.17–0.69	0.61

*BMI* body mass Index, *CAD* coronary artery disease, *CCB* calcium channel blockers, non-dihydropyridine, *CHF* congestive heart failure, *CI* confidence interval, *COPD* chronic obstructive pulmonary disease, *HR* heart rate, *PPG* photoplethysmography, *PVD* peripheral vascular disease, *MI* myocardial Infarction, *y* year

^a^We had 142 observations for model 1, owing to the inclusion of step counts and BMI as a predictor. The adjusted *R*^2^ was 0.01; *P* = 0.001

^b^We had 30,700 observations for model 2. The adjusted *R*^2^ was 0.009; *P* < 0.0005

Participants who reported having at least one medical condition (*n* = 27,958) contributed two thirds (2,007,418) of the HR-PPG measurements. They had a higher HR-PPG compared with those who reported no medical conditions (79.6 ± 14.2 bpm vs 77.6 ± 14.6 bpm, *p* < 0.0005), even after adjusting for age. Those suffering from any medical condition, except coronary artery disease, prior myocardial infarction (MI) and hypercholesterolemia had a significantly higher HR-PPG than those without the condition (Table 3). Once adjusting for age, HR was higher in those with hypercholesterolemia and coronary artery disease. The highest difference was observed in those with diabetes (82.6 ± 14.1 bpm vs 78.3 ± 14.5 bpm without diabetes), followed by those with COPD (82.5 ± 13.9 bpm with vs 78.3 ± 14.5 bpm without COPD). In multivariable analysis (Table 4, Model 2), female gender, all races/ethnicities other than non-Hispanic White and participants suffering from hypertension, hypercholesterolemia, diabetes, arrhythmia, sleep apnea, COPD, and asthma were independent predictors of a higher HR-PPG, whereas increasing age was a predictor of a lower HR-PPG. All medical conditions were associated with a higher HR-PPG, when adjusting for age, gender, and beta-blocker use (Supplementary Table 6). Similarly, asthma and COPD were associated with a higher HR-PPG after adjustment for beta-agonist use.

## Discussion

As the use of smartphone sensors and wearable devices provides data on cardiovascular parameters such as HR, physicians are increasingly expected to help patients interpret the results of these readings; however, existing norms derived from controlled, clinical settings may not reflect the range of HR values occurring in real-world conditions. Our validation demonstrated that smartphone-based HR-PPG strongly correlates with HR from the gold-standard ECG. This study provides the first and largest-scale description of real-world HR values derived from smartphone HR-PPG measurements from 66,788 individuals who provided over three million data points over a 3-year period. Furthermore, we describe how demographic and medical factors affect these norms, including age, gender, race/ethnicity, anthropometric characteristics, physical activity, and disease state. These data provide reference ranges of real-world HR for patients and physicians and establish the foundation for future research, in which real-world HR might become an outcome for large-scale studies to understand the evolution of disease at an international scale.

Higher baseline HR has been shown to be an important prognostic factor, with higher HR associated with increased all-cause and cardiovascular mortality.^1–5,10^ However, previous studies have shown that HRs measured in clinical settings may not be representative of real-world HR and may be biased by the particular clinical conditions for which the ECGs were ordered.^1,3^ For example, a “white-coat” effect can increase HR,^11^ leading to false elevation. Furthermore, ambulatory, real-world HR, has been found to correlate significantly more with mortality than resting heart rate obtained in the clinical setting,^12^ suggesting the need to update HR norms to reflect real-world, remotely obtained values.^6^ The median HR-PPG of 77.6 in healthy individuals of our cohort was higher than the median HR of 68.0 bpm described by Mason et al.,^3^ who studied 79,743 ambulatory subjects that had a single ECG done in a clinical setting. As we averaged across multiple measurements per user (median of 60.0 measurements per user per year), our data may provide a better approximation of the average real-world HR-PPG compared with a single measurement. The NHANES study followed 20,749 Adults living in the United States and described their in-clinic resting HR over 3 years.^2^ Compared with this study, the corresponding levels of the 5th percentile were lower in our cohort (50–55 bpm vs 60 bpm), which demonstrates a discrepancy between HR obtained in-clinic versus in a real-world setting, whereas in clinic HR tend to be trending higher than real-world HR.^2^ Whereas our 95th percentile was similar among those < 40 years old (104 bpm in both cohorts), among those > 40 years old, we found a lower 95th percentile (100 bpm vs 104 bpm),^2^ which may be due to our repeat measurements taken outside of the clinic setting, decreasing variability, and minimizing any “white-coat” heart rate effect.^11^ Furthermore, we described the circadian evolution of real-world HR, which allows us to interpret these values according to the time of day.^13^ We observed that HR-PPG and HRV levels decline with age. Our observations suggest that the 95th percentile of real-world HR-PPG is ≤110 in individuals aged 18–45 years old, ≤100 in those aged 45–60 and ≤95 bpm in individuals >60 years old. This decrease in maximum HR-PPG and HRV as people get older is mainly owing to a sympathetic modulation decline with aging.^10,14,15^

A considerable number of epidemiologic studies have demonstrated a link between a higher HR and increasing burden of atherosclerosis^16^ and cardiovascular outcomes^1,3–5,17,18^ as well as the existence of a biological gradient between the severity of atherosclerosis and resting HR.^19^ Increased HR has been linked to atherosclerosis risk factors and endothelial dysfunction, plaque erosion and plaque rupture.^20^ Furthermore, it is acknowledged that the stress on the cardiovascular system is better investigated by real-world measurements rather than measurements obtained at rest, in a stressful clinic environment.^6,12^ Real-world HR is more reproducible than resting HR obtained in clinical setting.^21^ Therefore, it is possible that real-world measurements would better correlate than resting HR measurements with cardiovascular outcomes.^12^ Our study observed a higher HR-PPG for participants with hypercholesterolemia, hypertension, diabetes, MI, a prior stroke and peripheral vascular disease, all risk factors or manifestations of systemic atherosclerosis.^1^ Similarly, a chronic increase in sympathetic tone leading to higher HR, as was observed in our cohort, has been described in patients with COPD, sleep apnea and asthmatic patients.^22–25^

In our cohort, women had a higher HR than those of men by 6 bpm, which extends prior observations to the real-world setting.^2,14,26–29^ It has been speculated to be owing to women having, on average, smaller stroke volumes.^1,26,28,30^ Differences in resting HR-PPG by race has also been previously reported in smaller studies of fewer than 170 participants.^22,23^ Bathula et al.^31^ demonstrated that on average, South Asians have 5 bpm higher HR-PPG than Europeans, findings that seemed genetically driven and were not related to other risk factors. Our cohort extends prior literature, by demonstrating within a larger sample size that African Americans had the highest HR-PPG. These racial differences may be explained by distinct genetic phenotypes, leading to a different neural control of HR-PPG in African Americans compared with Non-Hispanic Whites.^14^ We also observed an increase in HR-PPG and a reduction of HRV with increasing BMI, where individuals with a BMI ≥ 30 had a higher HR-PPG compared with their “normal weight” counterparts. These data reveal that obesity is associated with higher HRs, suggesting that weight loss may lead to lower HR and better overall health.^18,32^ Large-scale epidemiological studies involving 13,761 adults, demonstrated the link between an activation of the sympathetic nervous system, increased HR, and pulse pressure and BMI.^32^ Furthermore, we observed a “U-shaped” relationship with BMI and HR, where both underweight and overweight participants demonstrate an increase in HR compared with their ‘normal weights’ counterparts, complementing prior findings from the literature.^33^ We detected a reduction in HR with height, whereas the taller the person, the lower the heart rate was, extending prior findings from the literature.^34^ Our large sample enabled us to describe the real-world HR-PPG distribution according to daily step count strata. We observed that individuals with a higher activity level as measured by step counts had a lower HR-PPG and a higher HRV, which is consistent with prior studies.^35–37^ We also showed that for an increase of 5000 steps, the average resting HR-PPG decreased by 1 bpm, up to ~8000 steps/day. However, step count was not a significant predictor of reduced HR-PPG after multivariable adjustment, suggesting that the benefits of increased step counts might be difficult to disentangle from the effects of age, gender or racial differences. Our findings extend prior findings by being the largest cohort of real-world HR measures to date, reinforcing the notion that individual characteristics such as age, gender, ethnicity, step counts, and BMI should be taken into account when interpreting HR values in the clinical setting. Using repeated, real-world, HR-PPG data obtained from wearables or apps data could enable physicians to provide personalized HR goals to a level that was before unattainable.^9^

In this study, we have shown that HR-PPG measurements are valid, and our nomograms of HR-PPG measurements obtained by patients remotely can now be interpreted by physicians, across a wide variety of patient phenotypes. These data can inform patients about physical fitness and could help providers offer counseling on lifestyle changes or provide overall encouragement and support based on these real-world HR norms.^9^

Our study has several important limitations. Our enrollment of individuals who downloaded the Instant Heart Rate app may be associated with higher socioeconomic status, technological awareness, and knowledge of elevated cardiovascular risk factors. Our validation cohort comprised of consecutive patients referred to the cardiovascular clinic differing from the general population, which could limit generalizability. However, our validation was purposefully designed to look at a broader spectrum of people who might use the app-based PPG for HR measurements, including more people with abnormal ECGs and cardiovascular disease in whom PPG might be expected to be less accurate. Despite this, we demonstrated a high validity of these measurements, in line with previously published literature.

The PPG in our data was obtained using a specific app and accuracy of measurement may vary based on different user interfaces to ensure adequate contact and signal processing algorithms that may occur in different PPG approaches. In addition, as users recorded HR-PPG measurements on demand, rather than being passively monitored, available HR-PPG do not reflect all possible real-world HR and our nomograms might not generalize to HR values measured passively by wearables. In addition, we did not have the context around the measurements (i.e., food intake, post exercise, palpitations, etc.), which may have influenced the HR values. For example, in the “known resting HR data set”, we observed an average HR 2.8 bpm higher than in our full HR data set. One plausible explanation for this finding is that patients might be measuring their HR at rest, while having palpitations, leading to a higher upper boundary of HR in this data set and dragging the average HR higher. However, our high number of measurements collected per user in the full HR data set, combined with our large cohort size was able to describe the variability of HR according to age, gender, race, or BMI. Although the relationship between HR-PPG and step count confirms prior literature, our absolute values of step count may be underestimated owing to non-carrying time of the smartphone.^36^ Therefore, our findings should be interpreted with caution, especially in those >8000 average daily step counts, which represent a very small subset of participants in our study. The Health eHeart Study population is less racially, ethnically and geographically diverse and of a higher socioeconomic status than the average United States population, so care must be taken in applying these results to other populations with different characteristics.^18,33^ However, our population is likely representative of participants who are most likely to use this kind of technology. Owing to the cross-sectional nature of our study design, we were unable to investigate incident disease states and its relationship with HR-PPG and this should be examined in future studies. In addition, although self-reports of medical diagnoses in the HeH study is reliable,^37^ it may suffer from recall bias and social desirability biases.

Using a unique, real-world cohort that is the largest of its kind, we were able to describe the distribution of real-world HR-PPG among patients by means of remotely measured, smartphone-based PPG measurements. Our findings add granularity to the distribution of HR in specific subgroups not previously described and may assist physicians to interpret remotely obtained, real-world, on-demand, HR-PPG values measured by patients across a wide variety of patient phenotypes and medical conditions.

## Methods

### Study design

We first performed an in-person validation study of the app to determine its accuracy in assessing HR-PPG. We then analyzed 3,144,332 HR-PPG signals from 66,788 participants obtained using the app as part of the Health eHeart Study in a cross-sectional population-based study.

### Smartphone-based PPG validation study

In order to validate the accuracy of the app-based HR-PPG signal, we simultaneously recorded a 10-second HR-ECG and HR-PPG in 50 consecutive participants referred to Cardiology clinic at UCSF, after 5 min of rest. Mean differences were computed between successive cardiac cycles of HR-PPG and HR-ECG, in milliseconds and bpm. Rhythms were classified as being normal (sinus rhythm) or abnormal and by their regularity. Irregular rhythms included atrial fibrillation, premature ventricular, or atrial contractions and atrial flutter with variable atrioventricular block.

HR-PPG measurements were obtained using the Instant Heart Rate app (Azumio inc), a popular application for measuring HR and a smartphone’s (any Apple© model or Android© phone model) camera and light.^38^ PPG recordings are obtained “on demand” by the user steadily applying the pulp of their finger on the smartphone camera and thus is an on-demand measurement, as opposed to passive measurements made by some wearables. Participants were free to measure HR at any frequency and time of day. The study team did not provide additional instructions on when to measure such HR measurements.

### Population study design

We performed a cross-sectional analysis of data obtained from 1 April 2014 and 30 April 2018 from consecutive participants enrolled in the Health eHeart (HeH) Study—a worldwide, internet-based, longitudinal eCohort. English-speaking adults, 18 years or order, with an email address were eligible to join.^39^ The Health eHeart Study participants complete online surveys relating to demographics, physical activity levels, medical conditions, and medications in order to allow for the collection of patient reported outcomes and allows for connecting devices and apps (such as those that count steps) to the study.^39^ The study was approved by the UCSF Institutional Review Board and informed consent was obtained from all participants. For the analysis of HR-PPG, we included all Health eHeart Study participants that recorded at least one HR-PPG measurement and connected their Azumio account to the Health eHeart Study.

Participants were actively recruited through a variety of campaigns at UCSF (through clinics and electronically delivered invitations) and by partner organizations (e.g., American Heart Association), and passively recruited through word of mouth and press releases. For the first data collection set (“eVisit”), participants were asked to answer questions regarding the basic demographics, previous medical history and medications. We calculated body mass index from self-reported weight and height and classified individuals as normal weight (BMI ≥ 18.5 – < 25), overweight (BMI 25–30) and obese (BMI ≥ 30). We derived the following medication classes based on medication survey answers: beta blockers, beta agonists, amiodarone and non-dihydropyridine calcium channel blocks (CCB).

### Data collection

Heart rate measures using PPG were obtained using the Instant Heart Rate (Azumio, Inc) smartphone app on either Android or iOS operating systems and the smartphone camera. Resultant changes in reflected light intensity are interpreted by an algorithm as pulsatile blood volume changes, which is then translated into HR. At least 15 seconds of PPG signal, sampled at 100–120 Hz, were collected. Signals were processed to identify the rising edge in order to identify beat to beat intervals and calculate an average HR over the recording interval (Supplementary Fig. 1). If the underlying rhythm was an arrhythmia (atrial fibrillation, atrial flutter, premature ventricular contractions, supraventricular tachycardia), we used the peak of each HR-PPG waveform instead of the rising edge. Although it is difficult to record accurate HR measures during physical exertion, HR measurements taken immediately after a physical activity are possible.

### Weighting of repeated HR measurements

To account for repeated measures, HR and 24-hour step counts were log-transformed to approximate normality and the geometrical mean for each participant was calculated.^40^ To obtain the weights, we used linear mixed models with random intercepts to estimate the ICC of the repeated log-transformed measures. Then the weight for each participant was calculated as $\left( {weight_i = \frac{{N_i}}{{1 + ICC(N_i - 1)}}} \right)$, where *Ni* is the number of repeated HR measures for participants *I*. The denominator of the weight represents the inflation of the variance of the participant-specific means owing to the correlation of the repeated measures. In a final step, the weights were normalized to sum to the number of participants. If the repeated measures were independent (ICC = 0), then participants would be weighted in proportion to their number of observations; at the other extreme, if the repeated measures were perfectly correlated (ICC = 1), then the geometric mean for each participant would be given equal weight. We then used weighted linear models to examine the independent correlates of geometric mean HR.

### Statistical analysis

Continuous variables are presented using mean ± standard deviation (SD) or median (interquartile range) and were compared using the *t* test, the Mann–Whitney test or one-way analysis of variance, as appropriate. Categorical variables are presented as frequencies (percentages) and compared using either Chi-square or Fisher’s exact tests. For our validation study, we estimated ICC for agreement between HR-ECG values obtained in clinic, using a 12-lead ECG, with the HR-PPG obtained using the Azumio app, at the heart rate level and at the signal level, by comparing averaged R–R intervals between both methods. We also used a Bland–Altman plot to assess agreement between the simultaneous HR-PPG and HR-ECG recordings.^41^

To clean the data, we excluded outliers defined as values of HR-PPG outside of the biologically plausible range of 20–220 bpm.^16^ Next, to better limit the data to values most likely to be true resting values (not affected by physical activity), we created a “known resting HR data set”. To do this, we restricted our HR-PPG measurements to participants who had accumulated between 10 and 25 steps during the 30 min prior to their HR-PPG measurements, assuming that >25 steps represented the lower limit of any exercise in the last 30 min, whereas <10 steps might reflect users who set the phone down while exercising. We excluded participants with a medical condition from this data set. For our analyses, we summarized the repeated HR-PPG measurements for each participant, within our “known resting HR data set” and the “full HR data set” using weighted geometric means. As a measure of dispersion of the geometric mean HR-PPG, we used 95% prediction intervals, accounting for both the standard error of the overall mean and the residual variation of the participant-specific geometric means. We used a non-parametric kernel regression method to create centile charts for heart rate and step counts with respect to age, gender, and step count.

In the subgroup with at least one medical condition, we used unadjusted and age-adjusted HR-PPG to examine the independent associations of comorbidities with geometric mean HR-PPG. Univariable linear regression models were fitted to describe the relationship between age, gender, race/ethnicity, body mass index (BMI), height, weight, step count, and number of medical conditions. Furthermore, two multivariable linear regression models were fitted to examine the associations between HR and demographics or comorbidities, after assessing for heteroscedasticity and multicollinearity between the variables. The first included age, gender, race/ethnicity, number of medical conditions, BMI and step count, and the second included age, gender, individual medical conditions, medication use (beta blockers, amiodarone, beta agonists, and CCB). In an exploratory analysis presented in the appendix, selected interactions between medical conditions and medications were also included. Furthermore, we calculated person-based HRV, in beats per minute, by deriving the standard deviation of R–R intervals of HR-PPG within users with more than two recordings. We then fitted two multivariable regression models to examine the relationship between HRV and age, gender, medical conditions, BMI, and step count. We also derived average values of real-world HR-PPG and HRV based on time of day, based on weekend vs weekdays and based on seasons.

Two-tailed *p* values < 0.01 were considered statistically significant, without further correction for multiple testing. Statistical analyses were performed using STATA 15.1 (College Station, TX) and python 2.7 with packages scientific python version 0.19.1, scikit learn version 0.19.0.

### Reporting summary

Further information on research design is available in the Nature Research Reporting Summary linked to this article.

## Supplementary information

Reporting Summary

Supplemental Material

## Data Availability

The data that support the findings of this study are available on request from the corresponding author (J.O.). The data are not publicly available due to them containing information that could compromise participant privacy/consent.

## Supplementary information

**Supplementary information** accompanies the paper on the *npj Digital Medicine* website (10.1038/s41746-019-0134-9).

## Code availability

Our code is available at https://github.com/robertavram-md/Real-world-HR-norms.
