# Supplementary material for: Real-world heart rate norms in the Health eHeart study
Source: NPJ Digit Med. 2019 Jun 25;2:58. doi: 10.1038/s41746-019-0134-9 (PMC6592896; doi:10.1038/s41746-019-0134-9)
Supplement: Supplementary file 2 — Supplemental Material [file 41746_2019_134_MOESM2_ESM.pdf]

Supplemental materials

Supplementary Table 1. HR-PPG and HRV distribution according to dataset

Datasets	Known resting HR dataset<br>N = 40,572	Full HR Dataset<br>N = 66,788
HR-PPG description		
Number of HR-PPG measurements, n	40,572	3,110,988
Number of HR-PPG measurements per user, per year, median (IQR)	4.3 (9.2)	60.1 (145.4)
Average geometric mean HR-PPG±SD	81.8±19.6	79.0±14.5
95% Percentile Interval for HR	(52.5 – 132.1)	(54.5 – 110.8)
Average HR on weekdays, bpm	-	79.1±17.6
Average HR on weekends, bpm	-	78.4±17.1
Average HR during spring, bpm	-	79.0±17.4
Average HR on summer, bpm	-	78.8±16.6
Average HR on fall, bpm	-	78.4±16.5
Average HR on winter, bpm	-	79.1±16.7
HRV description		
Average HRV, bpm	13.4±11.4	15.8±9.2
Average HRV between 12 am and 6 am, bpm	-	13.4±10.4
Average HRV between 6 am and 12 pm, bpm	-	14.9±10.6
Average HRV between 12 pm and 6 pm, bpm	-	14.1±10.2
Average HRV between 6 pm and 12 am, bpm	-	12.5±9.6
Average HRV on weekdays, bpm	-	13.7±10.7
Average HRV on weekends, bpm	-	13.3±10.4
Average HRV during spring, bpm	-	14.1±10.2

Average HRV on summer, bpm	-	15.0±10.2
Average HRV on fall, bpm	-	14.2±10.0
Average HRV on winter, bpm	-	14.1±10.0

Abbreviations: HRV: Heart Rate Variability; HR-PPG; Heart rate measured using a smartphone photoplethysmography.

Supplementary Table 2A. HR-PPG Percentiles according to age, in healthy individuals

AGE	95P	90P	75P	50P	25P	10P	5P
**18**	105.9	101.8	99.6	87.2	69.9	63.1	61.9
**19**	102.2	96.0	89.6	80.8	71.5	65.2	57.9
**20**	105.5	99.6	90.6	81.1	72.9	64.4	60.0
**21**	103.9	99.3	91.5	80.9	72.6	64.8	60.2
**22**	107.3	98.8	89.6	81.4	72.1	63.7	58.6
**23**	107.6	100.1	90.2	81.0	71.9	64.6	59.4
**24**	104.8	99.2	88.9	79.1	70.2	63.8	59.7
**25**	107.4	99.6	90.0	80.7	72.2	63.6	60.5
**26**	105.2	99.0	88.9	79.8	69.9	61.9	58.6
**27**	103.3	97.4	88.7	79.5	70.0	62.2	57.8
**28**	104.6	97.1	87.7	78.5	68.6	61.0	57.4
**29**	105.4	99.4	88.4	77.8	69.2	61.4	57.4
**30**	107.9	99.1	89.4	78.9	70.0	61.3	56.3
**31**	105.6	99.2	88.8	78.8	68.9	61.4	58.4
**32**	106.2	99.5	89.7	79.1	69.4	61.0	56.6
**33**	105.1	98.9	88.4	77.8	69.6	62.2	58.0
**34**	104.7	97.0	86.7	77.4	68.3	61.1	57.9
**35**	106.2	99.8	88.7	78.5	68.7	61.0	56.6
**36**	103.6	97.4	88.0	77.7	67.4	58.9	54.6
**37**	105.6	98.5	87.9	77.2	68.2	61.4	57.1
**38**	106.0	98.2	86.4	76.8	67.7	59.6	55.6
**39**	107.0	98.9	87.0	78.3	69.5	61.7	57.1
**40**	101.7	96.3	84.9	75.0	67.3	60.7	57.5
**41**	101.7	95.3	86.3	77.2	68.0	61.5	56.4
**42**	103.1	96.7	86.6	75.8	65.6	58.7	53.9
**43**	97.4	93.2	83.6	74.2	65.8	57.5	54.8
**44**	101.7	95.2	84.9	74.8	65.7	58.7	55.1
**45**	99.9	94.1	85.8	75.8	66.8	59.2	55.4
**46**	102.3	93.7	83.3	75.0	65.7	57.2	54.6
**47**	99.8	92.7	83.0	73.1	65.2	58.0	54.5
**48**	98.1	91.3	82.0	73.7	65.4	57.8	54.0
**49**	101.3	94.6	83.3	74.1	65.4	58.3	54.2
**50**	96.1	90.1	81.3	73.1	63.9	57.6	53.1

**51**	101.4	92.2	81.2	73.8	65.9	59.7	55.7
**52**	97.0	92.4	83.4	73.0	64.8	58.6	55.3
**53**	96.1	88.6	80.3	72.2	64.8	58.7	54.2
**54**	96.6	91.1	81.8	73.3	64.7	58.8	55.6
**55**	97.8	91.4	82.0	72.5	63.8	56.7	52.9
**56**	97.4	90.0	82.3	73.0	64.9	57.5	54.4
**57**	94.6	88.5	80.4	72.7	64.7	58.9	56.0
**58**	96.1	90.7	82.4	73.1	64.1	58.0	56.1
**59**	98.7	90.4	80.9	71.6	62.7	58.4	55.5
**60**	99.2	90.9	80.0	71.2	63.2	57.8	52.3
**61**	94.4	88.0	78.9	72.4	64.8	60.0	57.4
**62**	100.8	91.1	81.5	72.5	64.1	56.8	53.3
**63**	96.1	89.3	81.5	72.3	64.8	58.5	55.0
**64**	97.1	86.9	79.3	70.5	63.1	57.9	55.5
**65**	93.6	87.5	81.7	73.3	66.0	61.0	56.3
**66**	95.7	89.1	81.8	73.1	63.3	57.0	54.1
**67**	86.5	83.2	77.7	71.3	65.8	59.3	56.4
**68**	90.2	86.3	81.1	72.3	64.7	60.7	57.8
**69**	99.7	93.8	79.9	71.1	64.2	59.9	56.2
**70**	91.9	87.9	81.3	72.6	62.2	58.7	57.8
**71**	94.9	91.1	80.1	74.0	64.6	56.9	54.8
**72**	96.4	92.8	79.0	71.5	63.1	59.6	56.6
**73**	88.9	86.1	79.1	72.7	64.3	60.9	60.0
**74**	92.3	88.2	79.9	73.4	67.0	59.5	57.6
**75**	94.3	87.6	83.5	76.7	68.5	61.5	57.9
**76**	91.1	85.9	77.3	72.4	65.1	56.5	51.9
**77**	90.4	86.8	81.6	77.2	75.0	66.3	58.5
**78**	88.6	85.6	75.3	70.1	66.1	57.9	53.6
**79**	86.7	86.1	84.3	81.2	78.4	76.7	76.1
**80**	86.3	81.9	74.5	72.4	71.7	68.9	67.3

Supplementary Table 2B. HR-PPG Percentiles according to age, in healthy females

Age	95p	90p	75p	50p	25p	10p	5p
**18**	97.5	96.0	91.5	87.5	80.3	69.9	66.4
**19**	98.9	96.0	89.9	83.1	71.8	66.8	62.5
**20**	103.8	99.0	91.0	81.8	75.2	66.4	60.6
**21**	108.1	101.3	91.0	82.0	73.6	66.0	62.8
**22**	104.6	99.4	89.6	81.9	73.4	66.9	59.9
**23**	106.8	99.6	90.0	81.1	73.2	66.6	60.5
**24**	103.2	98.2	89.5	80.4	71.1	64.4	60.9
**25**	104.7	99.3	89.9	81.9	73.3	63.9	61.3
**26**	102.5	96.9	88.6	79.8	71.5	63.0	60.0
**27**	103.7	98.6	89.8	81.7	72.2	65.9	59.0
**28**	105.3	98.8	88.5	79.2	70.6	62.0	58.2
**29**	105.4	101.7	90.3	79.9	71.4	64.8	59.5
**30**	108.2	102.0	91.3	80.1	70.5	62.1	58.5
**31**	107.2	100.1	89.8	80.1	70.7	63.3	59.8
**32**	106.2	100.0	90.3	80.8	71.1	63.6	58.5
**33**	104.7	100.2	88.4	80.4	71.7	65.3	61.9
**34**	104.2	97.3	87.7	79.9	70.5	62.8	59.5
**35**	105.1	100.3	90.6	80.0	70.3	62.0	57.5
**36**	102.9	98.7	90.5	80.6	71.7	64.2	55.8
**37**	104.5	98.6	89.2	78.0	69.0	64.7	60.7
**38**	103.3	97.2	86.0	77.7	69.8	62.7	59.1
**39**	102.3	98.4	87.8	81.1	71.5	64.3	59.7
**40**	102.7	97.4	87.5	78.1	69.3	64.1	60.7
**41**	103.2	96.9	90.1	80.6	72.5	64.5	60.8
**42**	105.0	99.6	90.5	79.3	67.8	60.1	55.4
**43**	97.3	93.9	84.5	74.9	67.2	61.1	57.1
**44**	102.4	96.3	88.1	78.5	68.3	61.7	59.2
**45**	100.5	98.4	88.4	79.3	69.3	63.4	59.0
**46**	99.8	93.1	84.7	77.9	70.0	62.2	57.2
**47**	99.7	94.1	83.6	74.2	65.2	58.6	56.5
**48**	96.2	90.5	83.0	75.2	68.5	62.2	57.9
**49**	104.3	99.5	85.9	77.0	68.0	59.6	55.4

**50**	100.5	92.3	83.2	75.1	67.3	59.3	55.2
**51**	97.7	89.9	81.1	74.8	66.8	61.2	57.5
**52**	102.5	96.3	85.7	75.8	68.0	61.8	59.2
**53**	96.2	89.6	82.4	74.8	68.7	62.5	58.3
**54**	96.3	87.1	81.4	73.4	67.3	59.3	56.7
**55**	105.4	97.7	83.7	74.1	66.1	57.2	55.2
**56**	97.9	90.0	82.5	74.4	66.4	58.6	56.6
**57**	92.4	87.6	78.8	71.6	64.3	60.1	57.8
**58**	99.9	92.2	82.2	74.2	65.8	59.9	55.9
**59**	96.8	89.7	81.0	74.3	66.0	62.1	60.2
**60**	100.3	97.1	83.4	74.1	66.2	61.3	58.1
**61**	97.0	91.4	80.1	73.4	67.6	61.6	59.7
**62**	99.5	90.2	81.1	74.6	68.8	58.4	56.1
**63**	100.5	91.4	83.3	73.6	65.6	59.8	58.1
**64**	109.0	96.9	82.7	73.7	65.9	61.9	59.6
**65**	94.4	88.0	83.0	76.3	68.0	62.8	59.1
**66**	103.2	93.0	82.0	74.4	65.6	58.8	57.4
**67**	85.4	84.1	79.1	72.3	65.9	57.6	56.6
**68**	88.2	85.9	82.0	75.1	69.0	64.7	62.2
**69**	94.6	92.2	79.9	73.0	68.4	63.8	63.3
**70**	92.2	89.6	81.9	77.4	65.2	61.1	59.0
**71**	91.5	86.9	82.1	76.1	72.0	64.5	62.8
**72**	95.2	92.8	88.9	76.2	72.0	68.3	66.3
**73**	89.5	87.5	85.5	75.5	69.0	65.0	64.2
**74**	91.7	87.5	77.0	73.5	68.4	66.7	66.1
**75**	94.3	92.2	84.1	76.6	70.0	66.7	61.6
**76**	94.3	89.6	77.7	69.6	64.8	61.7	58.5
**77**	93.6	91.4	85.2	81.2	76.4	74.6	74.1
**78**	82.5	79.9	74.6	73.4	66.9	64.3	63.9
**79**	81.9	77.3	71.8	68.5	62.1	58.0	54.0
**80**	72.8	72.8	72.8	72.8	72.8	72.8	72.8

Supplementary Table 2C. HR-PPG Percentiles according to age, in healthy males

Age	95p	90p	75p	50p	25p	10p	5p
**18**	102.4	96.4	88.6	76.9	68.0	61.2	57.8
**19**	94.9	93.3	86.9	75.3	67.5	59.3	57.2
**20**	96.1	93.1	87.0	76.5	67.9	61.1	58.0
**21**	99.9	95.8	88.9	75.6	67.9	59.4	55.5
**22**	98.2	93.0	82.8	75.3	66.9	60.7	56.7
**23**	106.9	98.3	86.4	76.5	67.5	58.9	56.4
**24**	103.7	98.8	83.9	73.0	65.7	60.3	56.5
**25**	104.6	97.2	87.6	77.5	68.2	62.1	58.6
**26**	103.6	92.4	85.1	75.7	65.3	59.6	57.5
**27**	96.0	91.5	81.3	71.7	64.6	58.9	56.1
**28**	99.2	94.6	82.6	73.9	65.7	58.5	55.5
**29**	98.4	94.1	84.2	73.6	65.2	57.2	55.4
**30**	98.9	95.3	86.4	74.6	66.0	56.9	51.8
**31**	101.4	96.0	86.1	75.0	65.3	60.5	57.2
**32**	105.9	95.7	85.6	74.4	66.1	57.5	55.0
**33**	100.5	95.0	84.8	73.9	65.9	58.0	53.8
**34**	98.3	91.3	83.6	74.3	65.1	58.8	56.3
**35**	104.2	95.1	83.9	74.6	64.9	58.6	54.3
**36**	98.2	91.6	83.5	72.9	62.6	57.1	54.4
**37**	102.5	95.8	83.5	74.1	65.9	57.9	54.9
**38**	106.0	97.3	83.8	72.4	63.0	55.7	54.1
**39**	109.5	98.6	84.6	74.5	65.2	57.9	55.5
**40**	97.9	92.0	80.9	71.7	64.4	58.3	54.6
**41**	95.2	91.3	82.9	72.9	65.2	56.9	53.4
**42**	100.4	95.1	81.5	72.6	64.6	57.1	52.5
**43**	94.8	88.6	81.4	71.4	63.2	55.6	53.8
**44**	98.5	90.8	80.2	71.6	63.1	56.2	52.0
**45**	95.7	91.3	81.4	72.5	62.9	56.5	53.2
**46**	101.8	91.6	80.7	71.9	62.6	55.1	52.0
**47**	99.3	91.9	82.3	71.0	62.8	55.8	51.1
**48**	97.3	91.3	80.4	70.7	62.6	54.3	51.4
**49**	96.5	90.8	80.1	71.1	62.9	55.7	52.9
**50**	90.1	84.1	78.5	69.5	62.0	55.0	51.6
**51**	102.1	92.6	79.6	72.2	64.4	58.3	52.6
**52**	90.7	86.8	80.2	68.3	61.9	56.3	53.9
**53**	90.6	85.0	77.1	68.2	62.1	56.3	52.8

**54**	94.5	88.0	78.8	70.9	62.6	56.6	54.1
**55**	91.6	88.3	79.6	69.1	60.8	53.3	50.9
**56**	97.3	90.9	80.8	70.9	62.9	55.3	51.1
**57**	94.1	87.6	79.7	72.5	63.2	56.8	55.7
**58**	94.7	87.2	80.1	71.0	61.8	57.2	54.8
**59**	96.0	88.1	78.0	69.6	60.6	56.2	54.5
**60**	94.4	87.0	77.1	69.0	61.3	55.7	50.4
**61**	87.3	83.8	77.1	69.0	61.9	58.7	55.1
**62**	97.3	92.8	76.2	67.1	62.4	53.2	50.5
**63**	87.0	82.8	77.9	68.9	62.6	54.9	53.2
**64**	80.9	79.8	72.3	65.5	60.2	56.0	54.5
**65**	91.5	85.2	78.5	69.2	63.7	56.2	53.8
**66**	88.8	85.1	78.2	69.4	61.9	55.7	49.9
**67**	81.5	79.2	76.1	69.1	65.0	59.4	55.4
**68**	87.8	85.6	77.5	69.7	61.9	58.2	54.8
**69**	102.5	88.5	72.3	68.4	64.2	58.5	55.1
**70**	90.3	86.8	79.1	69.6	61.4	58.2	56.3
**71**	92.7	90.2	77.6	69.0	62.1	55.3	54.9
**72**	79.2	77.8	73.1	64.2	61.2	56.8	55.3
**73**	88.1	84.4	74.7	68.2	61.5	60.0	55.5
**74**	85.2	82.4	76.5	69.6	58.8	55.9	54.4
**75**	92.0	88.0	83.3	75.4	68.6	67.6	64.9
**76**	84.0	80.3	74.0	68.4	58.4	50.7	50.2
**77**	83.9	82.7	79.1	76.9	71.9	63.1	60.2
**78**	92.3	89.3	70.1	67.2	58.8	52.9	50.2
**79**	86.7	86.1	84.4	81.4	78.5	76.7	76.1
**80**	88.8	87.0	81.5	72.4	72.4	72.4	72.4

Supplementary Table 3. HR-PPG Percentiles according to average daily step count strata

DAILY STEP COUNT	95P	90P	75P	50P	25P	10P	5P
**100 - 2000**	104.5	97.5	88.2	**79.3**	70.5	63.2	59.5
**2001 - 4000**	103.3	96.4	87.8	**78.9**	70.5	63.1	58.9
**4001 - 6000**	101.3	95.5	86.3	**77.8**	69.4	62.1	57.5
**6001 - 8000**	105.5	97.2	86.8	**77.1**	68.7	61.6	58.0
**8001 - 10000**	106.3	98.1	87.0	**76.0**	66.4	59.9	56.7
**10001 - 12000**	107.4	97.8	88.4	**76.9**	67.9	60.0	57.0
**12001 - 14000**	114.7	97.3	87.6	**78.9**	68.3	59.6	57.5
**14001 - 16000**	100.8	97.8	86.6	**76.4**	70.3	63.7	62.0
**16001 - 18000**	101.5	98.3	80.8	**75.1**	68.7	58.6	55.8
**18001 - 20000**	104.3	101.9	85.2	**76.5**	67.6	63.7	61.3

Supplementary Table 4. Baseline Characteristics for the known resting HR dataset and the full HR dataset restricted to those with no reported medical conditions.

Baseline characteristics	Known resting HR dataset [a,b] N=3,040	No reported medical conditions [a,b] n, (%) n=25,408
Age, mean±SD, yrs	41.6±13.0	37.7±13.0
Number of HR-PPG values recorded	14,364	1,103,570
Number of HR-PPG measurements per user, median (IQR)	3.0 (1.0 – 5.0)	17.0 (6.0 – 45.0)
Geometric HR-PPG, mean±SD, bpm	81.8±19.6	77.6±14.6
**Demographics**	**N=2,412**	**N=18,858**
Females	1,120 (46.4)	9,476 (50.2)
Males	1,292 (53.6)	9,382 (49.8)
**Race or ethnic group**	**N=2,412**	**N=18,727**
Non-Hispanic White	1,742 (72.2)	14,280 (76.3)
Black or African American	56 (2.3)	314 (1.7)

Hispanic, Latino or Spanish origin or ancestry	267 (11.1)	2,001 (10.7)
Asian	183 (7.6)	1,160 (6.2)
Multi-ethnic	109 (4.5)	526 (2.8)
Other	55 (2.3)	446 (2.4)
**Anthropometric data**	**N=480**	**N=2,175**
Height, mean±SD, m	1.73±0.10	1.73±0.10
Weight, mean±SD, kg	80.0±18.2	77.9 ±17.8
BMI, mean±SD, kg/m^2	26.6±5.4	26.1±5.3

Abbreviations: BMI: Body Mass Index; HR: Heart rate; m: meters; PPG: Photoplethysmography; SD: Standard deviation.

[a] Participants contributed measurements to both datasets.

[b] The distribution of all variables shared between the "known resting HR", the "No reported medical conditions dataset" are significantly different (p<0.0005), except the BMI (p=0.04), the "height" (p=0.22) and the weight (p=0.01) .

Supplementary Table 5. Univariable Predictors of Mean Heart Rate

Variable	Coefficient	95% CI	p-value
Age (10-year increments)	-1.75e02	-1.80e02 – -1.70e02	<0.0005
Gender, for females	4.44	4.15 – 4.72	<0.0005
Height, per cm	-0.23	-0.269 - -0.19	<0.0005
Weight, per kg	0.006	-0.01 – 0.03	0.54
BMI, per kg/m^2	0.25	0.25 – 0.26	<0.0005
Number of steps (1000-step increment)	-0.21	-0.42 – 0.01	0.05
**Race or ethnic group**			<0.0005
Non-Hispanic White	Ref	-	-
Black or African American	2.76	-0.60-6.12	0.11
Hispanic, Latino or Spanish origin or ancestry	1.08	-0.51 – 2.67	0.18
Asian	5.22	3.12 – 7.32	<0.0005
Multi-ethnic	2.66	0.07 – 5.24	0.04
Other/prefer not to disclose	0.44	-0.51 – 2.67	0.18

Number of medical conditions, per medical condition	0.65	0.33-0.96	<0.0005

Abbreviations: cm: centimeters; kg: kilogram;

Supplementary Table 6. Beta-blockers and beta-agonists multivariable regression models

**Model 1.** Multivariable regression describing the relationship between age, gender, beta-blockers use, hypertension and heart rate*			
Variable	Coefficient	95% CI	p-value
Gender, for females	4.17	3.85 – 4.47	<0.0005
Interaction between Beta-blockers use & hypertension	-4.50	-5.73 - -3.28	<0.0005
Beta-blockers use	2.66	1.75 – 3.58	<0.0005
Age, per 10y	-1.81	-1.93 – -1.70	<0.0005
Hypertension	3.76	3.36 – 4.16	<0.0005

**Model 2.** Multivariable linear regression describing the relationship between age, gender, beta-blockers use, hypercholesterolemia and heart rate *			
Variable	Coefficient	95% CI	p-value
Gender, for females	4.11	3.80 – 4.42	<0.0005
Interaction between Beta-	-2.43	-3.64 – 1.23	<0.0005
Age, per 10y	-1.79	-1.90 – -1.67	<0.0005
Hypercholesterolemia	2.70	2.33 – 3.08	<0.0005
Beta-blockers use	-2.43	-3.64 – -1.23	<0.0005

**Model 3.** Multivariable linear regression describing the relationship between age, gender, beta-blockers use, diabetes and heart rate*			
Variable	Coefficient	95% CI	p-value
Gender, for females	4.05	3.75 – 4.36	<0.0005
Interaction between Beta-	-4.21	-6.12 – -2.31	<0.0005

Beta-blockers use	1.32	0.68 – 1.96	<0.0005
Age, per 10y	-1.69	-1.80 – -1.58	<0.0005
Diabetes	7.02	6.28 – 7.77	<0.0005

Model 4. Multivariable linear regression describing the relationship between age, gender, beta-blockers use, CAD and heart rate*

Variable	Coefficient	95% CI	p-value
Interaction between Beta-	-4.71	-6.31 - -3.11	<0.0005
Gender, for females	4.04	3.73 – 4.35	<0.0005
CAD	2.97	2.14 -3.80	<0.0005
Beta-blockers use	1.74	1.06 – 2.43	<0.0005
Age, per 10y	-159	-1.70 - -1.48	<0.0005

Model 5. Multivariable linear regression describing the relationship between age, gender, beta-blockers use, prior myocardial infarction and heart rate *

Variable	Coefficient	95% CI	p-value
Gender, for females	4.04	3.73 – 4.35	<0.0005
Interaction between beta-blockers use & prior MI	-4.22	-6.35 - -2.10	<0.0005
Prior MI	3.08	1.81 – 4.36	<0.0005
Beta-blockers use	1.41	0.77 – 2.06	<0.0005
Age, per 10y	-1.57	-1.68 - -1.46	<0.0005

Model 6. Multivariable linear regression describing the relationship between age, gender, beta-blockers use, presence of arrhythmia and heart rate *

Variable	Coefficient	95% CI	p-value

Gender, for females	3.80	3.49 – 4.11	<0.0005
Interaction between beta-blockers use & arrhythmias	-0.53	-1.82 – 0.76	0.42
Arrhythmias	2.16	1.66 – 2.65	<0.0005
Beta-blockers use	0.89	0.15 – 1.63	0.02
Age, per 10y	-1.53	-1.64 – 1.42	<0.0005

Model 7. Multivariable linear regression describing the relationship between age, gender, beta-blockers use, peripheral vascular disease and heart rate *

Variable	Coefficient	95% CI	p-value
Gender, for females	4.04	3.73 – 4.35	<0.0005
PVD	3.25	1.64 – 4.87	<0.0005
Beta-blockers use	1.21	0.60 – 1.83	<0.0005
Age, per 10y	-1.56	-1.67 - -1.45	<0.0005
Interaction between Beta-blockers use &	-2.50	-5.97 – 0.97	0.16

Model 8. Multivariable linear regression describing the relationship between age, gender, beta-blockers use, stroke and heart rate *

Variable	Coefficient	95% CI	p-value
Gender, for females	4.03	3.71 – 4.33	<0.0005
Stroke	2.32	1.15 – 3.50	<0.0005

Beta-blockers use	1.02	0.40 – 1.64	<0.0005
Age, per 10y	-1.57	-1.68 - -1.46	<0.0005
Interaction between beta-blockers use & [illegible]	2.00	-0.77 – 4.78	0.16

Model 9. Multivariable linear regression describing the relationship between age, gender, beta-blockers use, CHF and heart rate *

Variable	Coefficient	95% CI	p-value
CHF	4.71	2.85 – 6.57	<0.0005
Gender, for females	4.03	-3.72 – 4.34	<0.0005
Beta-blockers use	1.19	0.56 – 1.82	<0.0005
Age, per 10y	-1.56	-1.67 – -1.45	<0.0005
Interaction between Beta-blockers use & CHF	1.19	0.56 – 1.82	<0.0005

Model 10. Multivariable linear regression describing the relationship between age, gender, beta-agonists use, asthma and heart rate *

Variable	Coefficient	95% CI	p-value
Gender, for females	3.94	3.64 – 425	<0.0005
Age, per 10y	-1.49	-1.59 – -1.38	<0.0005
Asthma	2.45	1.93 – 3.00	<0.0005
Beta-agonists use	4.20	0.66 – 7.73	<0.0005

Interaction between Beta-agonists use & asthma	-2.48	-7.91 – 2.94	<0.0005

Model 11. Multivariable linear regression describing the relationship between age, gender, beta-agonists use, COPD and heart rate *

Variable	Coefficient	95% CI	p-value
COPD	4.52	3.54 – 5.50	<0.0005
Gender, for females	4.00	3.69 – 4.31	<0.0005
Beta-agonists use	3.35	0.61 – 6.09	<0.0005
Interaction between beta-agonists use & COPD	10.09	-2.38 – 22.56	<0.0005
Age, per 10y	-1.54	-1.65 – 14.3	<0.0005

Abbreviations: 10y: 10-year increments; CAD: Coronary artery disease; CHF: Congestive heart failure; COPD: Chronic obstructive pulmonary disease; CCB: Calcium channel blockers; HR: Heart rate; IQR: Interquartile range; MI: Myocardial Infarction; PVD: Peripheral Vascular Disease.

Supplementary Figure 1. Photoplethysmography waveform as measured by the Azumio Instant HR app.

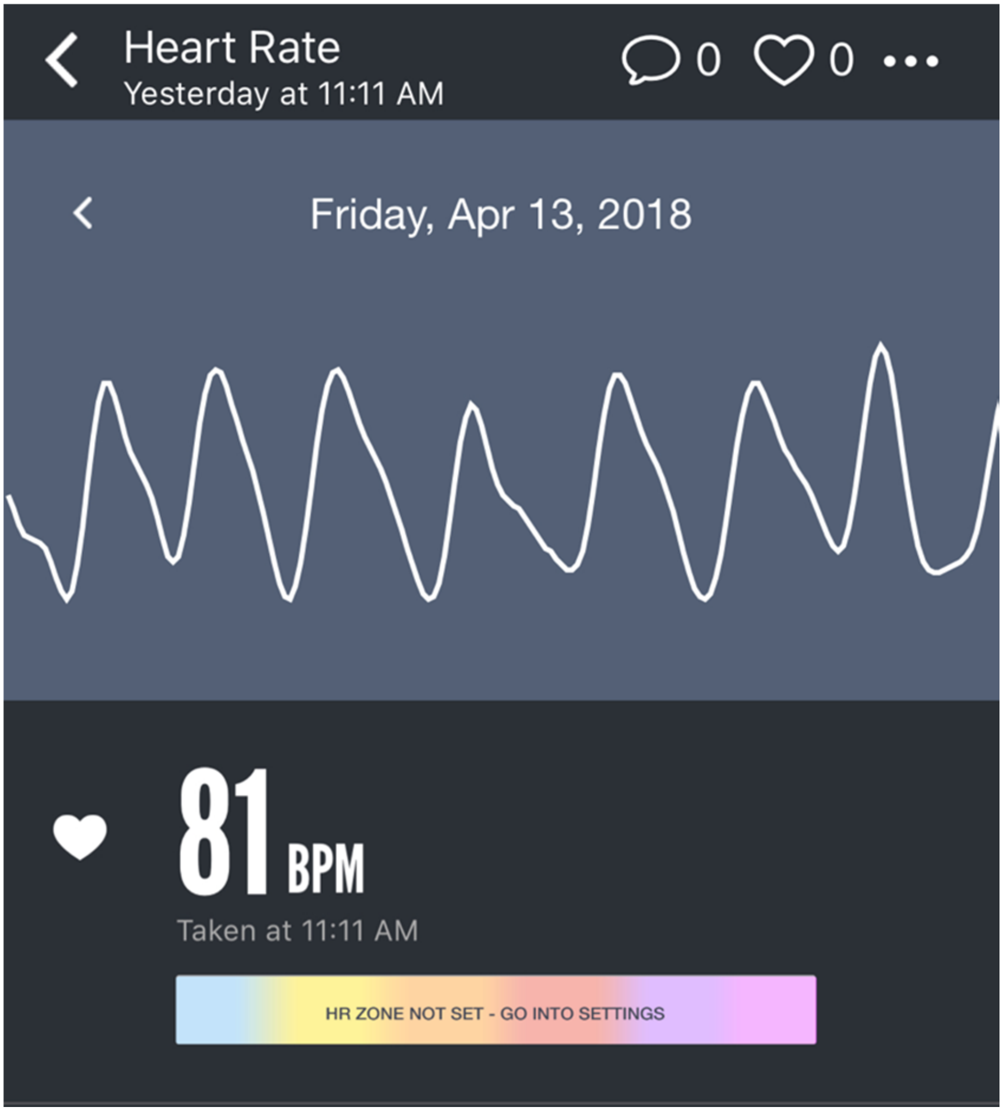

Supplementary Figure 2. Simultaneous HR-ECG recording and HR-PPG recording

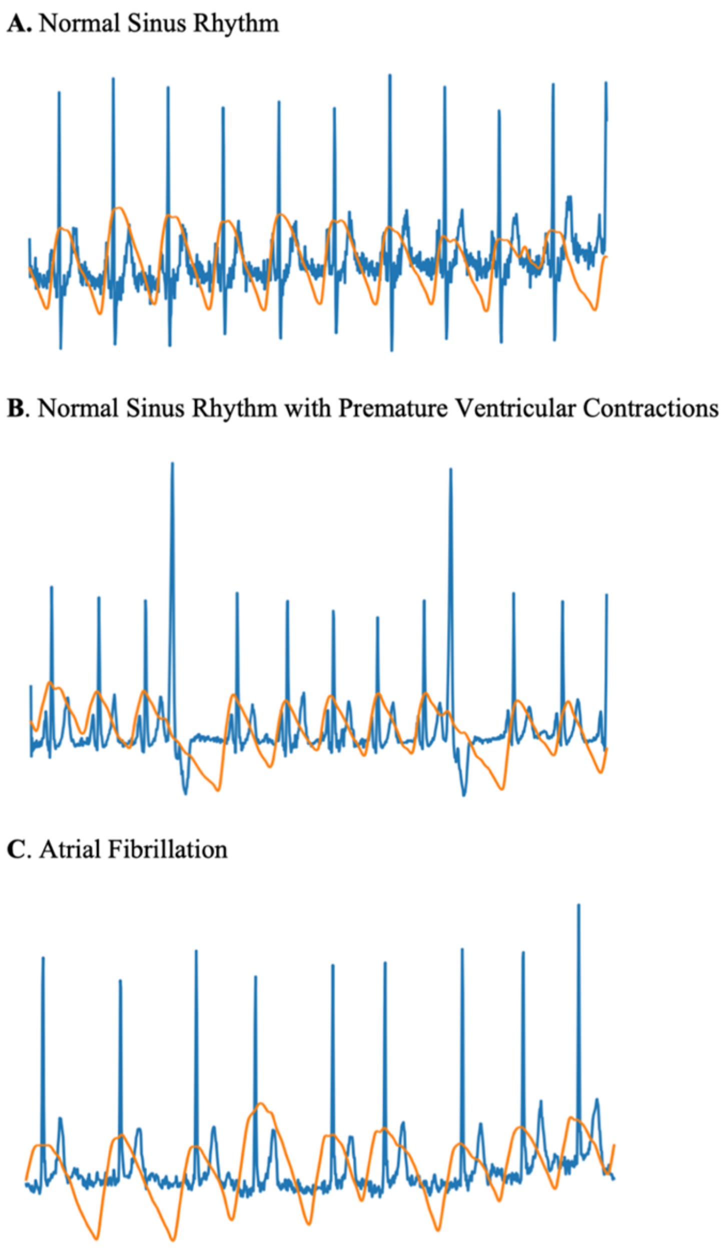

Blue waveform: HR-ECG recording; Orange waveform: HR-PPG recording.

Supplementary Figure 3

A. Bland-Altmann plot of RR intervals as measured by ECG and PPG, in milliseconds

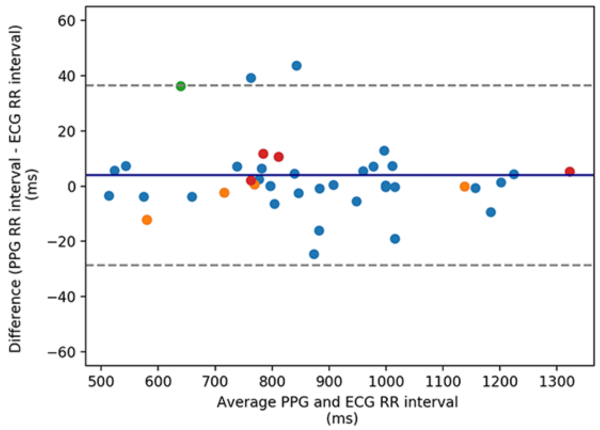

B. Bland-Altmann plot of average HR as measured by ECG and PPG, in beats per minute

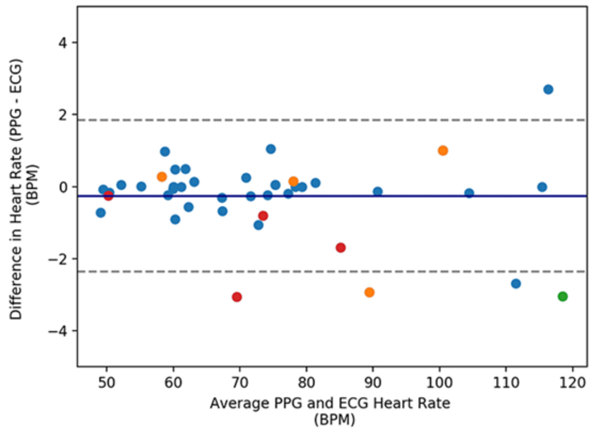

b.

Legend: Dashed lines: 95% confidence interval; Full blue line: mean values of the difference between PPG & ECG; Orange: Atrial Fibrillation; Red: Frequent PVCs; Green: Atrial Flutter: Blue dots: Normal sinus rhythm.

Supplementary Figure 4. Creation of our different datasets

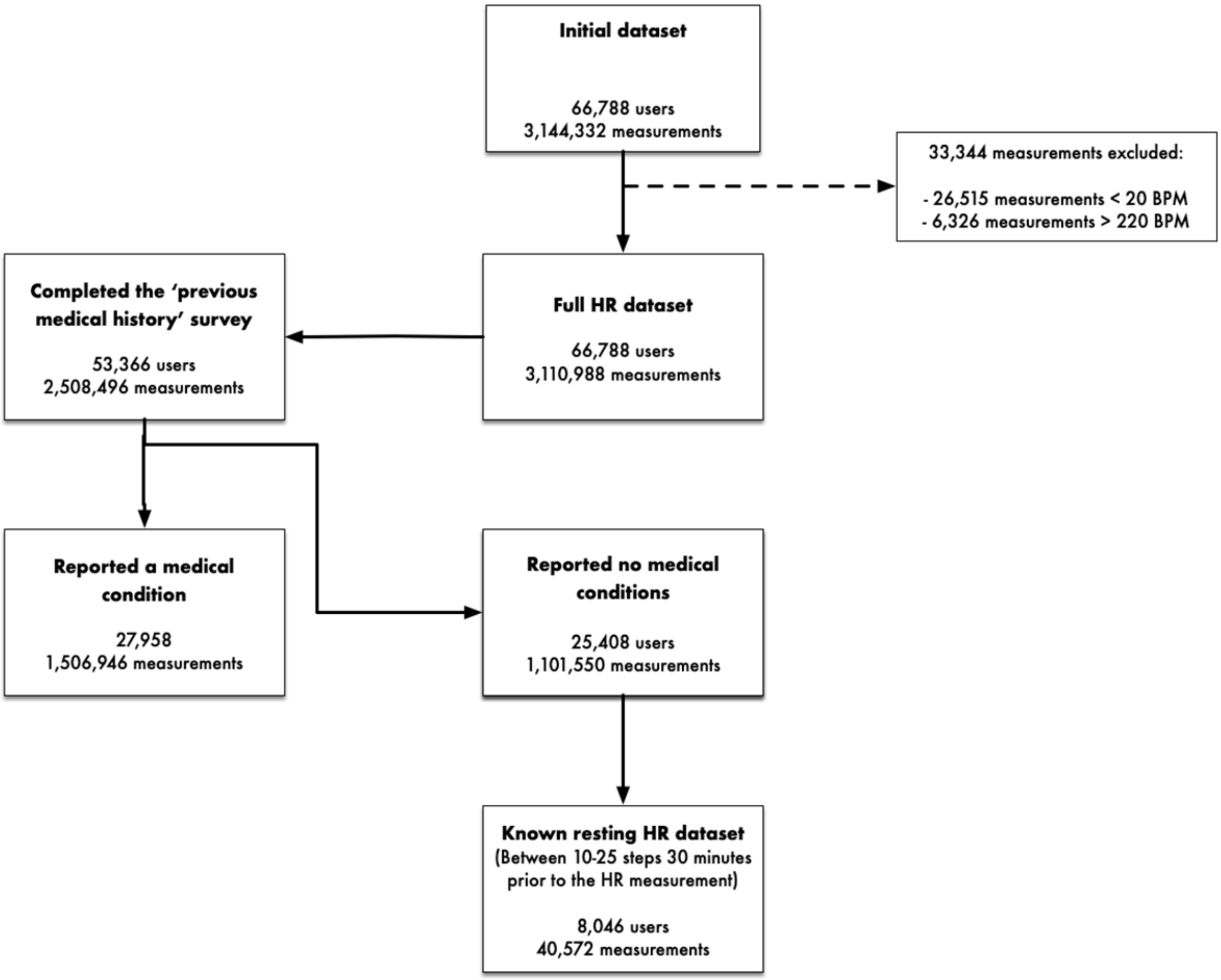

Supplementary Figure 5. Distribution of heart rate in our full heart rate dataset and in our known resting heart rate dataset

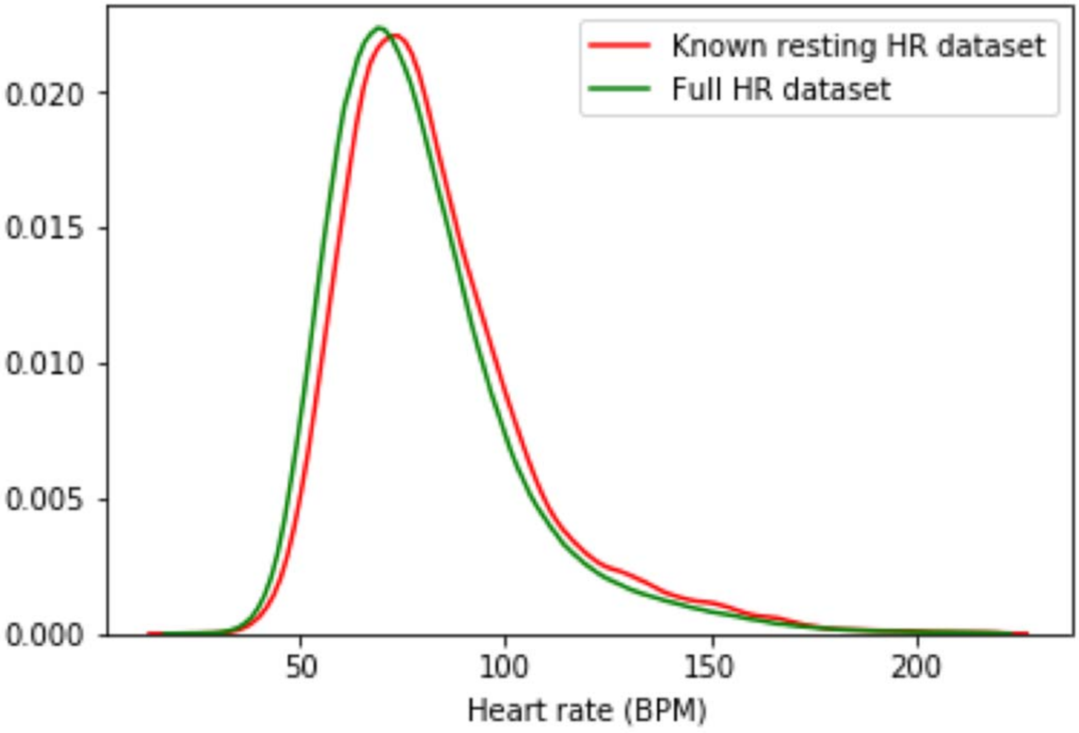

Supplementary Figure 6. Free-living HR difference from average according to the hour of the day

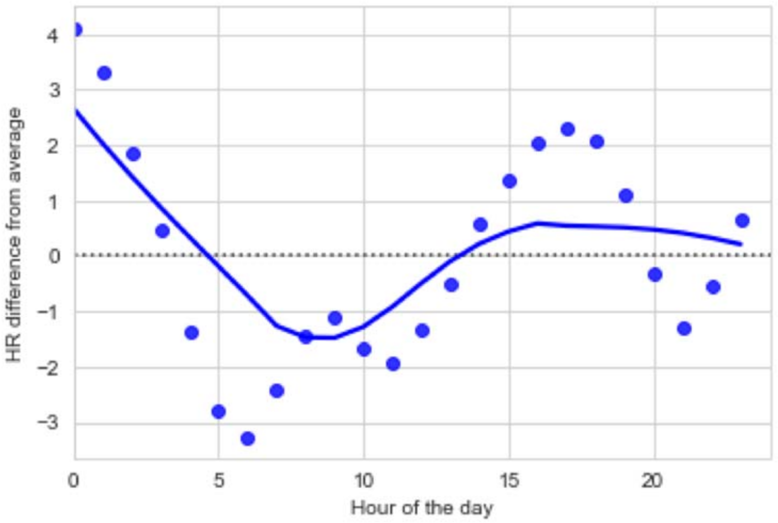

Hour	0	1	2	3	4	5	6	7	8	9	10	11	12	13	14	15	16	17	18	19	20	21	22	23
Number of recordings (thousands)	159	151	143	130	118	109	111	129	153	170	180	183	178	175	173	177	183	192	198	185	168	160	162	166
